# Risk factors and management of medical disputes: An analysis of preliminary appraisal reports

**DOI:** 10.1371/journal.pone.0334917

**Published:** 2025-11-10

**Authors:** Chu-Tzu Chang, Yung-Po Liaw

**Affiliations:** 1 Department of Public Health and Institute of Public Health, Chung Shan Medical University, Taichung, Taiwan; 2 Intellectual Property and Legal Affairs Office, Chung Shan Medical University Hospital, Taichung, Taiwan; 3 Department of Medical Imaging, Chung Shan Medical University, Hospital, Taichung, Taiwan; 4 Institute of Medicine, Chung Shan Medical University, Taichung, Taiwan; Tehran University of Medical Sciences, IRAN, ISLAMIC REPUBLIC OF

## Abstract

**Background:**

Medical disputes represent a growing challenge in healthcare, with implications for patient safety, legal liability, and institutional trust. Identifying contributing factors and risk patterns is essential for developing effective prevention strategies.

**Methods:**

We analyzed 70 preliminary medical dispute appraisal reports from Chung Shan Medical University Hospital (CSMUH), commissioned by Taiwan’s Ministry of Health and Welfare between 2017 and 2023. Descriptive statistics and logistic regression were used to examine demographic characteristics, institutional and specialty distributions, and associations between duty violations and malpractice determinations.

**Results:**

Most physician respondents were male (76.92%), while 56.16% of patients were female. Disputes were most frequently associated with medical centers (35.70%) and clinics (32.90%). In terms of specialty classification, surgical departments accounted for 55.29% of the specialties involved, including obstetrics and gynecology, orthopedics, and neurosurgery. Non-surgical departments accounted for 44.71%, including neurology, emergency medicine, and internal medicine. Violations of standard medical practice, incomplete documentation, and inadequate preoperative assessment were significantly associated with malpractice findings. Notably, inadequate preoperative assessment had an odds ratio (OR) of 39.74 (95% CI: 3.33–474.98, P = 0.0036), and disclosure failures had an OR of 12.75 (95% CI: 1.91–84.95, P = 0.0085).

**Conclusions:**

Duty violations related to clinical decision-making and informed consent significantly increase the likelihood of malpractice determinations. Targeted interventions in high-risk specialties and outpatient settings may improve legal defensibility and reduce preventable disputes.

## Introduction

Medical disputes are increasingly recognized not only as clinical challenges but also as legal and policy issues with direct implications for patient safety and institutional credibility [1,2]. These disputes often arise from disagreements over medical decisions, patient outcomes, and professional accountability. Despite growing attention, the underlying factors associated with malpractice determinations remain underexplored in Taiwan.

While some studies have examined overall trends in litigation and compensation, fewer have explored how specific institutional mechanisms—such as medical appraisal systems—affect legal outcomes. In China, forensic medical institutions play a critical role in determining liability and compensation in malpractice disputes. For example, one study found that 76% of claims resulted in financial compensation, and expert assessments influenced 93% of liability determinations. [2] Such findings highlight the powerful role of appraisal systems in shaping legal consequences, yet similar empirical analyses are lacking in Taiwan’s context. A more recent nationwide analysis further showed that selecting a judicial appraisal institution increased the likelihood of a favorable compensation judgment by approximately 10% and introduced marked inequities between litigants [3].

Medical malpractice litigation also imposes substantial psychological and professional burdens on physicians. Prior research shows that many physicians find the experience of being sued to be emotionally devastating, regardless of the outcome [4]. Notably, while only 3% of claims involve no harm, up to 37% of cases involve no actual error—yet legal proceedings still follow [5]. This discrepancy underscores the critical distinction between adverse outcomes and actual negligence, emphasizing the need for a system that accurately differentiates between the two.

Among cases confirmed for malpractices, violations of medical duty frequently stem from diagnostic errors, medical negligence, and surgical mishaps [6]. A recent nationwide study shows that diagnostic negligence—rather than the mere occurrence of an error—has become the primary focus of malpractice adjudication, especially in misdiagnosis-related claims [7]. In-depth analyses in the U.S. demonstrate that misdiagnosis remains one of the most frequent and harm-inducing causes of medical disputes [8]. Furthermore, judicial rulings in Taiwan frequently cite surgical errors—especially procedural complications and delayed diagnoses—as critical determinants of adverse outcomes [9]. Moreover, local analyses indicate that surgical specialties are disproportionately represented in cases involving diagnostic failures and adverse outcomes [9].

Misdiagnosis remains a leading cause of malpractice claims across various healthcare systems.

Studies have shown that diagnostic errors are often associated with severe outcomes, particularly in cases involving vascular events, infections, and cancer [8]. In the context of malpractice litigation, nearly 75% of severe misdiagnosis-related injuries are linked to these conditions [8].

Moreover, local analyses indicate that surgical specialties are disproportionately implicated in diagnostic failures and adverse outcomes [9].

Certain clinical environments also appear to increase litigation risk. Operating rooms and emergency departments, characterized by high patient turnover and time-sensitive decision-making, are commonly identified as the settings in which diagnostic and treatment errors most often occur [10,11]. These high-pressure environments pose systemic challenges, which may increase the likelihood of breaches in medical duty and, subsequently, the probability of malpractice allegations [11]. However, few studies have empirically analyzed how these risk factors interact with expert appraisal findings to shape legal outcomes.

In Taiwan, the number of medical dispute cases has shown considerable fluctuations, rather than a consistent upward trend. Assessments rose from 29 cases in 1992**–**221 cases in 2000, stabilized at roughly 200 cases in the early 2000s, and then declined to 119 cases by 2011 [10]. These trends indicate that the volume and nature of medical disputes are influenced by a complex interplay of factors, including systemic changes in healthcare delivery, evolving legal frameworks, and the effectiveness of dispute resolution mechanisms. To address these disputes, Taiwan established a centralized medical appraisal system administered by the Ministry of Health and Welfare (MOHW), wherein expert panels conduct independent reviews of medical records and issue structured written reports. Although the appraisal process is not open to the public and the full reports are generally not disclosed to litigants during ongoing litigation, these reports are frequently submitted to judicial authorities and serve as critical references in legal determinations and court rulings [12]. This expert-led, standardized mechanism represents a unique institutional approach to resolving medical disputes, yet little empirical research has evaluated how the content of these appraisal reports—particularly findings of duty violations—influences legal outcomes.

To address this gap, the present study examines a series of preliminary medical dispute appraisal reports commissioned by the MOHW and conducted at Chung Shan Medical University Hospital. Using structured content analysis and statistical modeling, this research aims to identify demographic, clinical, and institutional factors associated with appraised negligence. Specifically, it explores how different types of duty violations, medical specialties (grouped as surgical vs. non-surgical), and institutional settings (e.g., clinics vs. hospitals) influence malpractice determinations. The findings are intended to inform policy development, enhance risk management strategies, and support improvements in patient safety within Taiwan’s healthcare system.

## Materials and methods

### Data collection

This study utilized medical dispute appraisal reports issued by the MOHW of Taiwan following the Guidelines for Medical Dispute Appraisal [1 2]. These guidelines stipulate that medical dispute appraisals are conducted solely upon formal requests from judicial authorities and are conducted by the Medical Appraisal Panel under the MOHW Medical Affairs Review Committee. The appraisal process involves reviewing relevant case materials, obtaining a preliminary report from designated physicians, and a final determination by the panel. This ensures professionalism and impartiality in the issuance of a formal appraisal report under the MOHW’s authority.

### Study design

This study employed a retrospective cross-sectional design to analyze 70 preliminary medical appraisal reports commissioned by the MOHW and conducted at CSMUH between 2017 and 2023. The primary data sources included the appraisal reports and corresponding medical case records. The key variables extracted for analysis included the gender and age of defendants and patients, the level of healthcare institutions involved, types of litigation, patient treatment outcomes, and breaches of medical obligations. Notably, the first author participated in assisting with each preliminary identification report for the cases at CSMUH.

### Criteria for Data Selection

**Fig 1:**
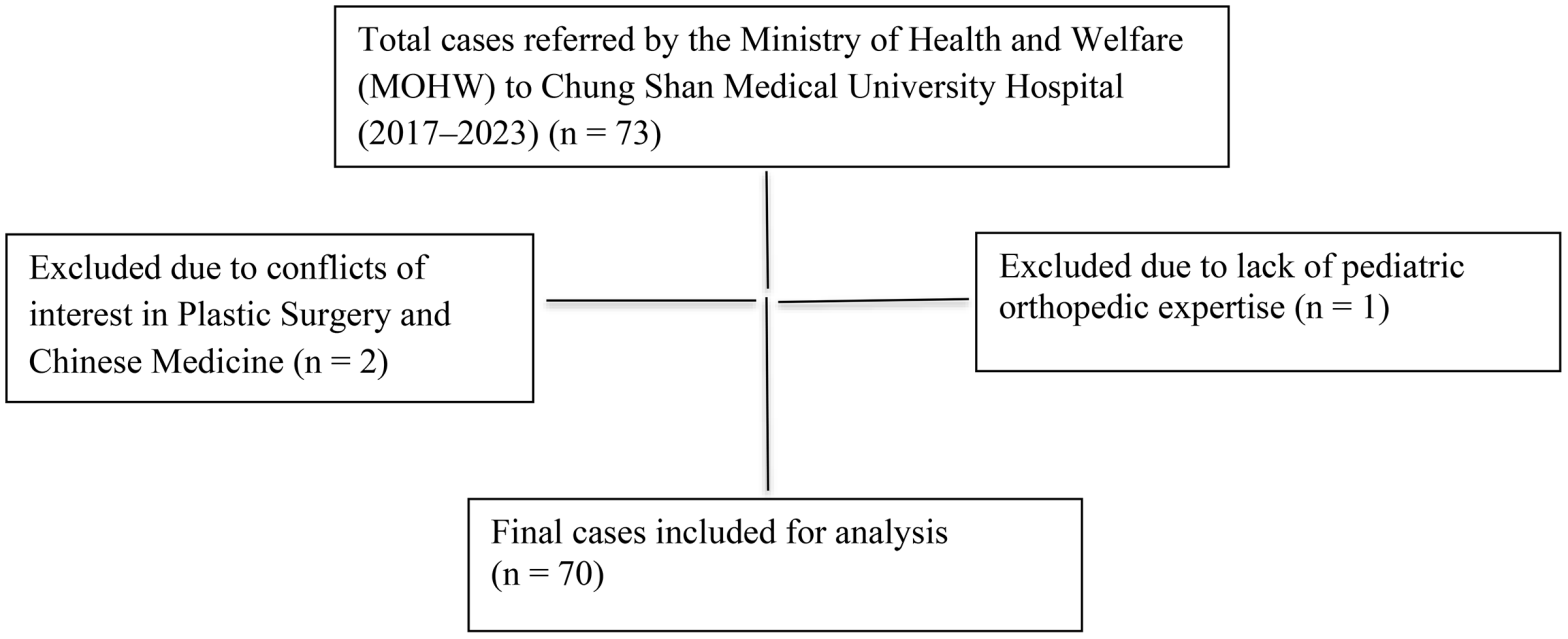
Flowchart of the medical dispute case selection process.

This study included appraisal reports submitted by the Ministry of Health and Welfare (MOHW) to CSMUH for expert evaluation. These reports were reviewed by experts designated by CSMUH and subsequently returned to the MOHW. Cases involving the same patient, physician, and medical institution but adjudicated at different judicial levels were treated as a single case for analysis. Exclusion criteria included instances where CSMUH declined appraisal due to conflicts of interest or a lack of relevant expertise. Two cases were excluded due to declared conflicts of interest by specialists in plastic surgery and traditional Chinese medicine. One additional case involving pediatric orthopedic surgery was excluded because CSMUH’s orthopedic specialists lacked experience with children under seven years old. After these exclusions, 70 cases were included in the final analysis (Fig 1).

### Appraisal report preparation and review process

Each preliminary appraisal report was prepared by a specialist attending physician designated by a medical center or quasi-medical center commissioned by the MOHW. Upon completion, the report underwent an initial review by a senior attending physician at the vice-president level.

It was then submitted to the Joint Commission of Taiwan (JCT) for verification, ensuring consistency between the case summary, appraisal conclusions, and supporting medical records.

If discrepancies were identified or the report failed to completely address the appraisal questions, it was returned to the physician for revision and resubmission. Once finalized, the report was forwarded to the MOHW Medical Review Committee for final assessment. The JCT functioned as an independent review body, providing external validation, providing external validation for the appraisal reports referenced in this study.

### Data collection methodology

The appraisal reports used in this study were anonymized by assigning serial numbers before analysis. The research team performed the initial coding and categorization of the data, and cases with missing data were excluded from the analysis.

### Statistical analysis

Data were analyzed using SAS v8.3. Logistic regression—which is well suited to binary (and multinomial) outcomes and allows simultaneous adjustment for multiple covariates—was applied, with a recent practical example provided in Fuente-Mella et al. [13] and detailed methodological guidance in Harrell [14] and the SAS/STAT User’s Guide [15]. We employed logistic regression analysis to examine associations between independent variables and the outcome variable—determinations of malpractice. Both univariate and multivariate logistic regression analyses were performed, and adjusted ORs with 95% confidence intervals (CIs) were reported. Variables with P < 0.05 in univariate analysis were entered into the multivariate model using a stepwise selection method. We additionally used linear regression to explore continuous secondary outcomes (e.g., appraisal turnaround time) [14,15].

### Statistical parameters

The following statistical parameters were used in this study: central tendency and dispersion indicators, including mean, median, interquartile range (IQR), and 95% CI. A significance level of P < 0.05 was considered statistically significant. All estimates were reported with 95% CI to assess the precision and variability of the statistical results.

### Regression models and variable definitions

In the linear regression analysis presented in Table 5, the dependent variable was the number of violation items identified per case, treated as a count-based continuous variable. The main independent variable was the appraisal outcome, categorized into three groups: Malpractice, Cannot Determine, and the reference group, No Malpractice. Additional covariates included the number of undetermined items in each case and the total number of disputes. These covariates were included to control for potential confounding effects and to enhance model robustness.

**Table 5:** Linear regression analysis of violation counts.

Variable	Unadjusted Model			Fully Adjusted Model		
	Beta	95% CI	P-value	Beta	95% CI	P-value
Malpractice						
No	—	—	—	—	—	—
Yes	5.66	4.70–6.62	<0.0001	5.61	4.64–6.58	<0.0001
Could not Determine	1.74	0.88–2.60	0.0002	2.50	1.25–3.74	0.0002
Undermined Violation Count	—	—	—	-0.25	-0.54–0.05	0.1066
Total Dispute Count	—	—	—	0.01	-0.12–0.14	0.9067

Note: Table 5 results are presented for unadjusted and fully adjusted models. The adjusted model includes Undetermined Violation Count and Total Dispute Count as continuous covariates. Beta coefficients represent the estimated difference in the number of violations relative to the No Malpractice reference group. CI = Confidence Interval.

In the logistic regression analysis presented in Table 6, the dependent variable was whether the appraisal outcome indicated medical malpractice. Independent variables included three main categories: (1) specific violations of medical duties such as failure to disclose, deviation from standard practices, and incomplete medical records; (2) institutional-level variables such as whether the case originated from a clinic; and (3) departmental characteristics, specifically whether the involved specialty belonged to a surgical or non-surgical category. The surgical/non-surgical classification was introduced as a binary variable to examine the association between procedural complexity and malpractice findings. Surgical departments were defined based on standard specialty classification and the nature of procedural involvement. All predictors were entered simultaneously into the model to assess their adjusted effects on the likelihood of a malpractice determination.

**Table 6:** Logistic regression analysis of the risk of being appraised as negligent.

Risk of Being Appraised as Negligent	Beta	P-value	OR	95% CI
Reference (no obligation Violation) Risk of Malpractice determination Violation of disclosure Obligation	2.55	0.0085	12.75	1.91–84.95
Violation of medical practice standards	4.97	< 0.0001	144	11.29 – > 999.99
Side Effects Related to Treatment	3.30	0.0041	26.99	2.84–256.38
Incomplete medical records Documentation	2.26	0.0124	9.55	1.63–55.99
Violation of preoperative Assessment Obligation	3.68	0.0036	39.74	3.33–474.98
Delayed treatment	16.05	0.9806	>999.99	<0.0001 –>999.99
Incorrect treatment	25.01	0.9679	>999.99	<0.0001 –>999.99
Compared to non-clinic Institutions: risk of malpractice determination				
Clinic	1.94	0.0297	6.96	1.21–40.07
Surgical specialty	0.77	0.1293	2.16	0.79–5.75

Note: Table 6 presents the odds ratios (ORs) and 95% confidence intervals (CIs) for variables associated with the likelihood of receiving a negligence determination in medical appraisals. Statistical significance was assessed at an alpha level of 0.05.

### IRB approval and ethical considerations

This study is a retrospective analysis based solely on anonymized medical records analyzed anonymized medical records derived from previously issued appraisal reports. No direct contact with human subjects or additional clinical interventions were involved. The study protocol was reviewed and approved by the Institutional Review Board of Chung Shan Medical University Hospital [IRB approval number: CS1–24130]. All procedures were conducted following ethical standards, including the Declaration of Helsinki and ICH-GCP standards. Given the retrospective nature of the study, which involved only fully anonymized data without identifiable personal information, the requirement for obtaining informed consent was waived by the IRB..

## Results

As shown in Table 1, most medical dispute cases in Taiwan between 2017 and 2021 were appraised as involving no negligence. On average, 86.94% of cases each year fell into this category, with the highest rate in 2017 (89.00%). In contrast, the proportion of cases determined as negligence averaged 4.39% across the five years, ranging from 2.54% in 2017 to a peak of 6.65% in 2018. The rate of possible negligence remained low throughout the study period, with a five-year average of 2.51%, peaking at 4.30% in 2021 and dropping to 0.77% in 2018. In contrast, most of the medical dispute cases were concluded as no negligence, with annual proportions consistently exceeding 84%. The highest rate was recorded in 2017 (89.00%), and the overall average across the five years was 86.94%. These findings indicate that confirmed or suspected medical negligence was relatively rare, and the majority of appraisals did not support malpractice claims.

**Table 1:** Statistical summary of medical dispute appraisal results by Taiwan’s Ministry of Health and Welfare.

Appraisal Results	2017	2018	2019	2020	2021	Average
Negligence	9 (2.54%)	26 (6.65%)	17 (4.56%)	15 (4.21%)	10 (3.58%)	15.4 (4.39%)
Possible negligence	4 (1.13%)	3 (0.77%)	14 (3.75%)	11 (3.09%)	12 (4.30%)	8.8 (2.51%)
No negligence	315 (89.00%)	345 (88.24%)	318 (85.25%)	311 (87.36%)	235 (84.23%)	304.8 (86.94%)
Indeterminate negligence	18 (5.08%)	13 (3.32%)	20 (5.36%)	19 (5.34%)	18 (6.45%)	17.6 (5.02%)
Others	8 (2.26%)	4 (1.03%)	4 (1.07%)	0 (0.00%)	4 (1.44%)	5 (1.43%)
Total	354 (100%)	391 (100%)	373 (100%)	356 (100%)	279 (100%)	350.6 (100%)

*Note: Others include non-medical disputes, incomplete appraisals, and other categories* [16]. Source: https://www.mohw.gov.tw/lp-121-2.html

Table 2 summarizes the demographic, institutional, and litigation-related characteristics of 70 medical dispute appraisal cases evaluated at CSMUH from 2017 to 2023. Female patients comprised a slightly higher proportion than males (56.16% vs. 43.83%), with a median age of 49 years (IQR = 25), compared to 44 years (IQR = 30.5) for males. In contrast, most defendants (76.92%) and appraisal panel members (81.57%) were men, reflecting a notable gender imbalance among healthcare providers and evaluators.

**Table 2:** Background of medical dispute appraisal cases (n = 70).

Category	Number	Percentage (%)	Age (Median)	IQR
Patient				
Men	32	43.83	44	30.50(Q1 = 27.5, Q3 = 58)
Women	41	56.16	49	25 (Q1 = 37, Q3 = 62)
Defendant				
Men	80	76.92	–	–
Women	24	23.07	–	–
Appraisal Physician				
Men	62	81.57	–	–
Women	14	18.42	–	–
Institution type of defendants				
Medical Centers	25	35.70	–	–
Regional Hospitals	16	22.90	–	–
District Hospitals	5	7.10	–	–
Clinics	23	32.90	–	–
Nursing Homes	1	1.40	–	–
Treatment outcome				
Death	27	38.57	–	–
Injury	43	61.42	–	–
Type of litigation				
Criminal	27	38.57	–	–
Civil	24	34.29	–	–
Civil and criminal	19	27.14	–	–

Note: Age statistics are reported only for patient groups using medians and *interquartile ranges* (*IQR*).

Medical centers (35.70%) and clinics (32.90%) were the most frequently involved institutions, while district hospitals and nursing homes were rarely implicated. Regarding clinical outcomes, 61.42% of cases involved patient injuries, and 38.57% resulted in death. Nearly two-thirds of cases involved criminal or combined civil and criminal litigation, underscoring the legal seriousness of the disputes. Specifically, 38.57% of cases were criminal, 34.29% civil, and 27.14% involved both types. These characteristics suggest that institutional type, patient outcomes, and litigation severity may interact with how appraisal panels assess negligence—issues explored further in the following analysis.

Table 3 summarizes the medical specialties of the defendants involved in the 70 medical dispute appraisal cases analyzed in this study. A total of 85 specialties were identified because some appraisal cases involved multiple specialties being simultaneously subject to litigation or appraisal, resulting in a higher total count of departments than the number of individual cases.

**Table 3:** Medical specialties of defendants involved in medical dispute appraisal cases (n = 85 specialties across 70 cases).

Category	Specialties Included	Number	Percentage (%)
Surgical	Obstetrics and gynecology, Orthopedics, Neurosurgery, Plastic surgery, Colorectal surgery, Urology, Ophthalmology, Otorhinolaryngology, General surgery, Dentistry	47	55.29%
Non-surgical	Neurology, Thoracic medicine, Cardiology, Chinese medicine, Dermatology, Emergency medicine, Pediatrics, Rheumatology, Anesthesiology, Diagnostic radiology, Nursing, Other (Psychiatry, Family medicine, Nephrology, etc.)	38	44.71%
Total	–	85	100%

Note: A total of 85 specialty entries were recorded across 70 medical appraisal cases. Some cases involved multiple specialties. Specialties were grouped into surgical and non-surgical categories based on primary clinical roles. Surgical departments included those where procedural interventions are routine (e.g., obstetrics, orthopedics). Non-surgical departments primarily focus on diagnostic and medical management (e.g., neurology, internal medicine).

To enhance interpretability, specialties were grouped into surgical and non-surgical categories. Surgical specialties accounted for 55.29% of involved departments (n = 47), with obstetrics and gynecology, orthopedics, and neurosurgery among the most frequently cited. Non-surgical departments comprised 44.71% (n = 38), including neurology, cardiology, internal medicine, and emergency medicine. This distribution suggests that while medical disputes span a broad range of specialties, surgical departments are disproportionately represented, possibly due to the higher procedural risks inherent in invasive interventions.

Table 4 presents the appraisal outcomes and dispute categories from 70 medical dispute cases evaluated at CSMUH between 2017 and 2023. The majority of cases (77.14%) were assessed as involving no malpractice, whereas 10.00% were found to involve malpractice. In 12.86% of the cases, the panel was unable to determine whether malpractice had occurred. The most prevalent dispute types included surgical disputes (68.57%), diagnostic disputes (61.43%), and examination-related issues (60.00%). Other commonly observed categories were medication-related disputes (51.42%), postoperative care (50.00%), infection (50.00%), and preoperative evaluation (35.71%). Less frequent, yet noteworthy, were disputes concerning medical equipment (34.29%), referral or consultation (30.00%), emergency care (27.14%), and anesthesia (12.85%). These findings underscore the clinical diversity and complexity of medico-legal assessments observed in this study. Such diversity is consistent with prior research, which found that malpractice disputes frequently span multiple clinical domains and involve overlapping categories [10]. Notably, dispute categories are not mutually exclusive, as individual cases often involve multiple issues simultaneously (e.g., both surgical and diagnostic errors). Therefore, cumulative percentages exceed 100%. This reflects the multifactorial nature of clinical disputes and should be considered when interpreting descriptive patterns.

**Table 4:** Summary of violations in medical appraisal cases.

Case results and types	Number (n = 70)	Percentage (%)
Appraisal results		
No malpractice	54	77.14
Malpractice	7	10.00
Cannot determine	9	12.86
Dispute Types		
Examination dispute	42	42/70 (60.00%)
Surgical dispute	48	48/70 (68.57%)
Anesthesia dispute	9	9/70 (12.85%)
Medication dispute	36	36/70 (51.42%)
Medical equipment dispute	24	24/70 (34.29%)
Diagnostic dispute	43	43/70 (61.43%)
Preoperative evaluation	25	25/70 (35.71%)
Postoperative care	35	35/70 (50.00%)
Infection Dispute	15	15/70 (21.43%)
Referral/Consultation	21	21/70 (30.00%)
Emergency Care Dispute	19	19/70 (27.14%)

***Note:*** *Dispute types are not mutually exclusive; some cases involve more than one category.*

Table 5 presents the results of a linear regression analysis evaluating the association between medical appraisal outcomes and the number of identified violation items across 70 cases reviewed at CSMUH between 2017 and 2023. In the unadjusted model, cases appraised as involving malpractice were associated with an average of 5.66 more violation items compared to cases without malpractice (95% CI: 4.70 to 6.62, P < 0.0001). Additionally, cases classified as Cannot Determine had 1.74 more violations on average than those with no malpractice (95% CI: 0.88 to 2.60, P = 0.0002). In the fully adjusted model, which included Undetermined Violation Count and Total Dispute Count as continuous covariates, associations with appraisal outcomes remained significant. Malpractice cases showed an estimated increase of 5.61 violation items (95% CI: 4.64 to 6.58, P < 0.0001), and Cannot Determine cases had an increase of 2.50 items (95% CI: 1.25 to 3.74, P = 0.0002). The number of undetermined violations (β = –0.25, 95% CI: –0.54 to 0.05, P = 0.1066) and the total number of dispute items (β = 0.01, 95% CI: –0.12 to 0.14, P = 0.9067) were not statistically significant. These results suggest that appraisal conclusions—particularly the identification of malpractice—are robustly associated with a greater number of confirmed medical duty violations.

Table 6 summarizes the results of a logistic regression analysis evaluating risk factors associated with malpractice appraisal outcomes. Among individual violation types, failure to conduct adequate preoperative assessment (OR = 39.74, 95% CI: 3.33–474.98, P = 0.0036) and deviation from established medical practice standards (OR = 26.99, 95% CI: 2.84–256.38, P = 0.0041) were the strongest predictors of malpractice findings. Other significant predictors included failure to fulfill disclosure obligations (OR = 12.75, 95% CI: 1.91–84.95, P = 0.0085) and incomplete medical documentation (OR = 9.55, 95% CI: 1.63–55.99, P = 0.0124). At the institutional level, cases originating from clinics were significantly more likely to be appraised as malpractice compared to those from hospitals or other non-clinic settings (OR = 6.96, 95% CI: 1.21–40.07, P = 0.0297). Although delayed treatment and incorrect treatment were associated with extremely high odds ratios (>999.99), these results were not statistically significant, likely due to small case numbers or wide confidence intervals (P = 0.9806 and P = 0.9679, respectively). To explore the influence of medical specialties, a binary variable distinguishing surgical and non-surgical departments was included in the model. Surgical involvement was associated with higher odds of malpractice appraisal (OR = 2.16, 95% CI 0.79–5.75; P = 0.1293), but this association did not reach statistical significance.

## Discussion

This study found that violations of preoperative assessment, disclosure obligations, and standard medical practices were significantly associated with malpractice determinations. In addition, cases originating from clinics exhibited substantially higher odds of malpractice appraisal, and surgical specialties were disproportionately represented among confirmed negligence cases. Demographic patterns also revealed that male physicians were more frequently involved in disputes, while female patients comprised the majority of claimants, suggesting potential influences of clinical exposure and litigation behavior. Existing studies on medical disputes are limited in several key aspects. Most prior research has focused on litigation cases or individual reports, with insufficient use of comprehensive medical appraisal data to assess dispute characteristics and contributing factors [2,5]. Quantitative analyses exploring associations between physician gender, hospital level, and dispute types (e.g., diagnostic errors, surgical complications) remain insufficient [6,7,11]. Furthermore, cross-specialty comparisons are lacking, making it difficult to identify medical disciplines at higher risk for malpractice [9]. Few studies have examined whether specific breaches of medical duty—such as incomplete documentation or failure to follow standard procedures—significantly increase the likelihood of negligence findings [5]. These gaps highlight the need for research using appraisal-based data and robust statistical methods to better understand the determinants of medical dispute outcomes.

Our findings revealed that male physicians were more frequently involved in disputes than female physicians, consistent with previous reports indicating that male practitioners tend to face higher litigation risks and may practice more defensively in response to malpractice concerns [17]. In our cohort, 56.2% of claimants were female, suggesting either greater clinical exposure or a higher propensity to file formal complaints. The median patient age was 46 years, indicating that disputes predominantly involved middle-aged adults—possibly reflecting higher healthcare utilization and stronger awareness of legal rights in this demographic. For comparison, a Japanese study by Watari et al. reported a considerably lower median age of 33 years, highlighting cross-national differences in population structure, medical culture, and dispute behavior [7].

These demographic patterns may reflect broader trends in litigation behavior, as prior studies have noted that patient age and gender can influence perceptions of communication quality and decision satisfaction—factors that are known contributors to malpractice claims [17,19].

Further analysis identified a lack of informed consent and communication failure as major contributors to dispute outcomes. This is consistent with prior findings that cite inadequate disclosure and poor physician–patient dialogue as common bases for malpractice litigation [19,20]. Effective communication has been shown to mitigate litigation risks by fostering patient trust and reducing dissatisfaction, which is a known precursor to formal complaints [18]. Moreover, standardized consent protocols and shared decision-making practices have been associated with improved legal outcomes and reduced claim rates in high-risk clinical settings [18,19].

Analysis of institutional-level data revealed that clinics and medical centers were the most frequently involved facilities in the dispute cases. Clinics accounted for 32.90% of all cases, while medical centers comprised 35.70%. This finding may be explained by the high patient volumes, limited resources, and structural differences between these settings. Clinics often lack comprehensive risk management systems or formal legal counsel, making them more vulnerable to disputes—especially in cases involving documentation issues or communication breakdowns [18].

The logistic regression analysis further indicated that cases originating from clinics were associated with significantly higher odds of being appraised as malpractice (OR = 6.96, 95% CI: 1.21–40.07, P = 0.0297). This result underscores the need for targeted risk-reduction strategies in outpatient and community-based settings. Additionally, although cases involving surgical specialties showed higher likelihoods of being judged as negligent (OR = 2.16), this association did not reach statistical significance (95% CI: 0.79–5.75, P = 0.1293), possibly due to limited sample size or uneven specialty distribution. Nonetheless, this non-significant trend aligns with prior studies suggesting that surgical disciplines may be more frequently targeted in litigation due to procedural complexity and heightened patient expectations [5,7,9].

Cross-specialty analysis revealed that obstetrics and gynecology, neurology, orthopedics, and neurosurgery were among the most frequently cited fields in malpractice-related appraisals. These specialties are often associated with high-stakes decisions and irreversible outcomes, which may explain their disproportionate involvement in disputes. The findings align with previous research identifying these disciplines as having elevated litigation risk due to the complexity of procedures and patient expectations [6,9]. This diversity is consistent with prior research noting that malpractice disputes often span multiple clinical domains and overlapping categories [10]. Because multiple dispute types were often present in a single case, overlapping categories may introduce interaction effects or reduce the stability of certain estimates.

In terms of case severity, the mortality rate among the 70 disputes analyzed was 38.57%, underscoring the high clinical stakes involved in many of these cases. This relatively high death rate is consistent with previous findings indicating that fatal or permanent injuries significantly increase the likelihood of litigation and malpractice determinations [5].

Inadequate informed consent and poor physician–patient communication were recurrent themes across the appraisal reports. Consistent with previous findings, failures in disclosure obligations—such as insufficient explanation of treatment risks or lack of documentation—were significantly associated with malpractice determinations in our regression model [18,19]. Prior literature has underscored that patients who feel excluded from decision-making or uninformed about treatment options are more likely to perceive negligence and seek legal recourse [19].

This suggests that patient dissatisfaction stemming from poor communication may independently increase the incidence of disputes. Interventions such as shared decision-making tools, communication skills training, and the use of structured consent templates have shown effectiveness in reducing litigation risk [18,19]. In addition, a civil-court review of 946 closed malpractice verdicts in Taiwan revealed that only 14% of claims resulted in physician liability, despite prolonged litigation times, underscoring systemic inefficiencies and the importance of alternative dispute mechanisms [20]. From a policy perspective, strengthening communication competencies in medical education and continuing professional development programs may serve as long-term preventive strategies. Furthermore, institutions should consider implementing standardized consent procedures—especially in high-risk departments—to ensure clarity, patient understanding, and legal defensibility.

Given our limited sample size, the statistical power to detect smaller effect sizes may be reduced, and our results should be interpreted with caution, as they may not necessarily indicate the presence or absence of a true association. Despite these limitations, the study presents several key strengths. It utilizes appraisal-based data that are rarely examined empirically, drawing on preliminary medical dispute appraisal reports from Taiwan’s Ministry of Health and Welfare (MOHW). This unique dataset plays a crucial role in legal and administrative decision-making. Furthermore, the research adopts a multidimensional analysis that integrates clinical, institutional, and procedural variables, offering a comprehensive understanding of the factors associated with malpractice determinations, thus enhancing the overall insights into medical disputes.

## Conclusion

This study provides empirical insights into the characteristics of medical dispute cases in Taiwan, focusing on the content and implications of expert appraisal reports. Violations of preoperative assessment, disclosure obligations, and standard medical practice were identified as significant predictors of malpractice determinations. Moreover, clinics and surgical specialties were disproportionately represented among cases with confirmed negligence, underscoring the need for tailored risk mitigation strategies. By linking clinical, institutional, and procedural variables with appraisal outcomes, this study highlights critical leverage points for improving dispute resolution and enhancing medical safety. Policymakers and healthcare administrators should consider investing in standardized communication protocols, informed consent practices, and institutional support mechanisms—particularly in high-risk settings—to reduce preventable harm and foster trust in healthcare systems.
